# Supplementary material for: Silicon Nanowire Field-Effect Transistor as Biosensing Platforms for Post-Translational Modification
Source: Biosensors (Basel). 2020 Dec 21;10(12):213. doi: 10.3390/bios10120213 (PMC7767353; doi:10.3390/bios10120213)
Supplement: Supplementary file 1 [file biosensors-10-00213-s001.pdf]

Supplementary Materials

Table 1. Element contents on the modified surface.

Elements	Nude	APTES	APTES+GA	APTES+GA+PSGL-1
O1s	74.52%	68.05%	51.17%	29.08%
C1s	24.42%	28.78%	45.8%	60.85%
N1s	1.06%	3.17%	3.13%	10.06%

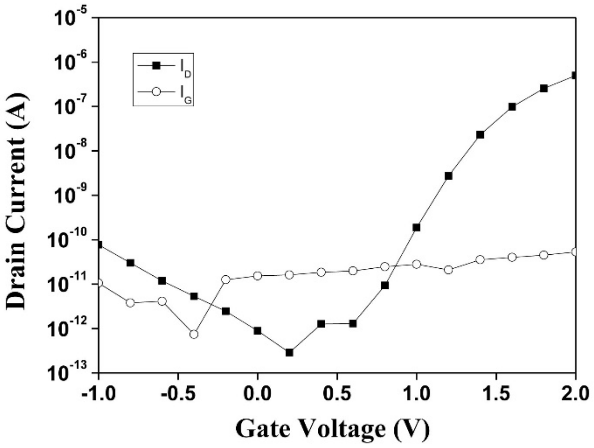

Figure 1. Electrical characteristics of the poly-Si NWFET.

I_D–V_G curve of the poly-Si NWFET device, the drain current (I_D) and gate leakage (I_G) were showed respectively.

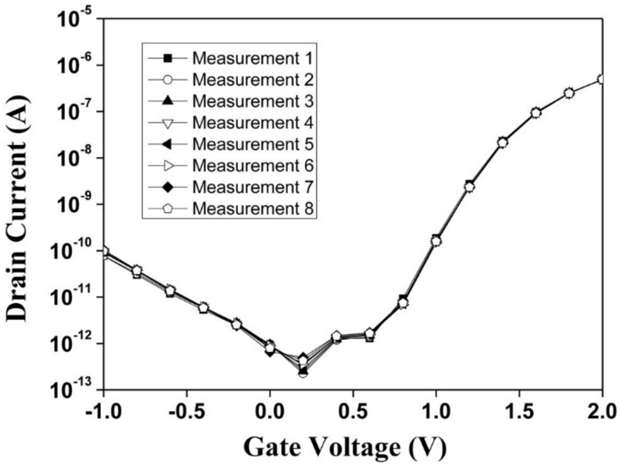

Figure S2. Eight consecutive measurements of I_D-V_G of the device

Eight consecutive measurements of the I_D-V_G curve are shown. Each of the I_D-V_G curves is close, indicating the stability of the device.

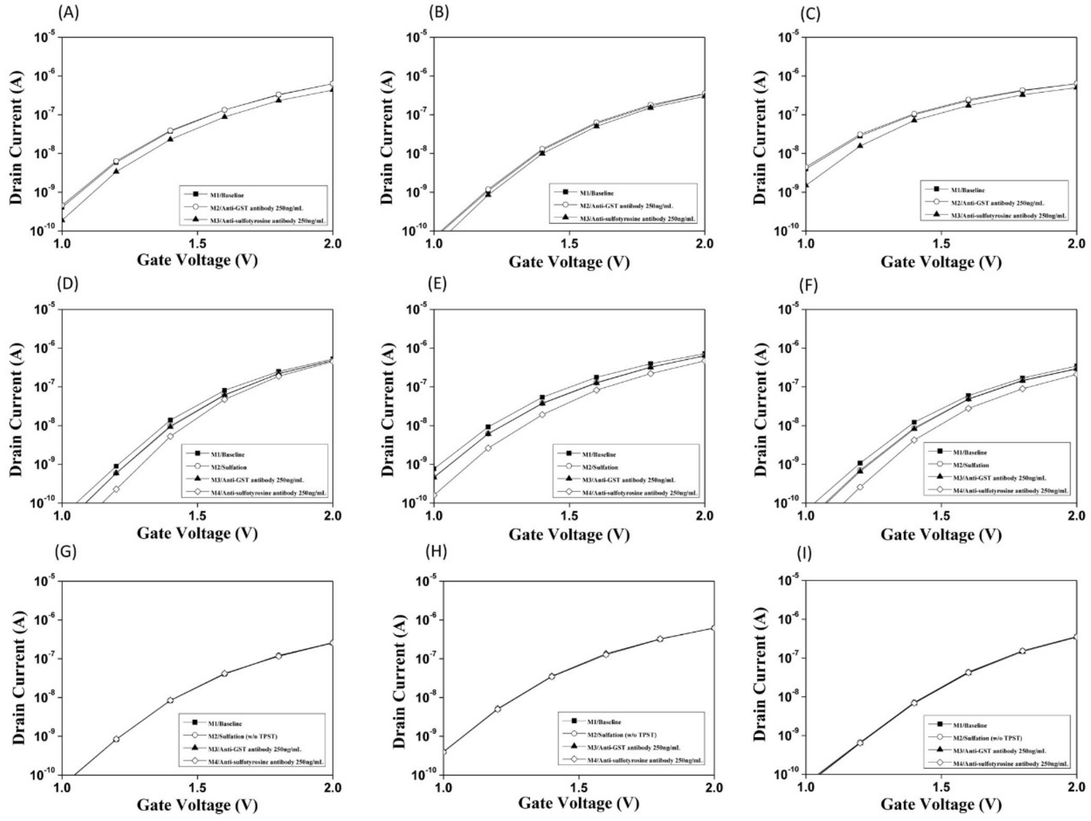

Figure S3. Electrical responses of the functionalized pSNWFET to PSGL-1 sulfation

(A)(B)(C) I_D–V_G curve obtained from the synthesized sulfated PSGL-1 peptide and interaction with anti-sulfotyrosine, and anti-GST antibodies as the controls. (D)(E)(F) I_D–V_G curve obtained through PSGL-1 sulfation and interaction with anti-sulfotyrosine, and anti-GST antibodies as the controls. (G)(H)(I) I_D–V_G curve obtained from non-sulfated PSGL-1 following coupled enzyme treatment without the critical enzyme TPST and the interaction with anti-sulfotyrosine, and anti-GST antibodies as the controls.

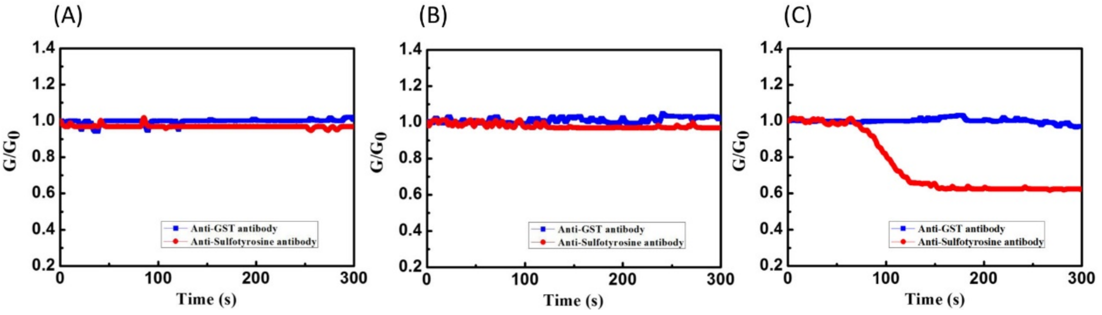

Figure S4. Real-time response of the pSNWFET following different surface modifications

(A) Response of the pSNWFET with an unmodified surface to anti-GST and anti-sulfotyrosine antibodies. (B) Response of the pSNWFET with a non-sulfated PSGL-1 peptide-modified surface to anti-GST and anti-sulfotyrosine antibodies. (C) Response of the pSNWFET with native PSGL-1 peptide-modified surface following PTS catalyzed by PST–TPST coupled enzyme system to anti-GST and anti-sulfotyrosine antibodies. The conductance was measured at fixed gate voltage. G_0 was the conductance

obtained from the anti-GST treatment as the baseline, and the changes in the conductance (G/G_0 = Ganti-sulfotyrosine/Ganti-GST) were calculated.

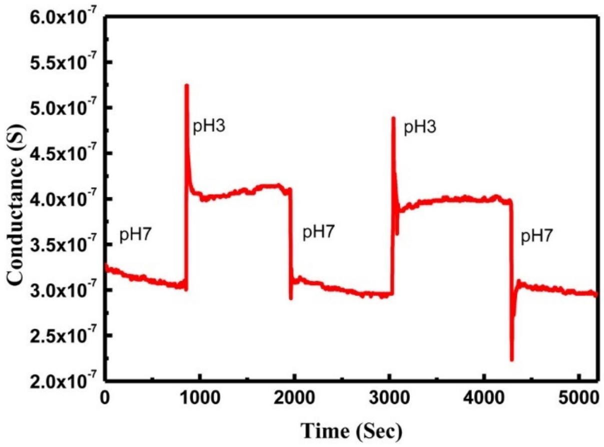

Figure S5. pH profile of the PSGL-1 modified surface

The real-time conductance response of the PSGL-1 modified NWFET device in 2 different pH buffers.
